# Muscle-Saturated Bioactive Lipids Are Increased with Aging and Influenced by High-Intensity Interval Training

**DOI:** 10.3390/ijms20051240

**Published:** 2019-03-12

**Authors:** Ditte Søgaard, Marcin Baranowski, Steen Larsen, Michael Taulo Lund, Cathrine Munk Scheuer, Carina Vestergaard Abildskov, Sofie Greve Dideriksen, Flemming Dela, Jørn Wulff Helge

**Affiliations:** 1 Xlab, Centre of Healthy Aging, Department of Biomedical Sciences, University of Copenhagen, 2200 Copenhagen N, Denmark; stelar@sund.ku.dk (S.L.); michaeltl@sund.ku.dk (M.T.L.); catrold@gmail.com (C.M.S.); carina.vestergaard.abildskov@gmail.com (C.V.A.); sgdideriksen@gmail.com (S.G.D.); fdela@sund.ku.dk (F.D.); jhelge@sund.ku.dk (J.W.H.); 2 Department of Physiology, Medical University of Bialystock, 15-089 Bialystock, Poland; marcin.baranowski@umb.edu.pl; 3 Clinical Research Centre, Medical University of Bialystock, 15-089 Bialystock, Poland; 4 Department of Geriatrics, Bispebjerg University hospital, 2400 Copenhagen NV, Denmark

**Keywords:** aging, ceramide, diacylglycerol, high-intensity interval training, insulin resistance, obesity

## Abstract

Ceramide and diacylglycerol are linked to insulin resistance in rodents, but in humans the data are inconsistent. Insulin resistance is frequently observed with aging, but the role of ceramide and diacylglycerol is not clarified. Training improves metabolic health and, therefore, we aimed to elucidate the influence of age and high-intensity interval training (HIIT) on ceramide and diacylglycerol content in muscle. Fourteen young (33 ± 1) and 22 older (63 ± 1) overweight to obese subjects performed 6 weeks HIIT three times a week. Maximal oxygen uptake and body composition were measured and muscle biopsies and fasting blood samples were obtained. Muscle ceramide and diacylglycerol were measured by gas-liquid chromatography and proteins in insulin signaling, lipid and glucose metabolism were measured by Western blotting. Content of ceramide and diacylglycerol total, saturated, C16:0 and C18:0 fatty acids and C18:1 ceramide were higher in older compared to young. HIIT reduced saturated and C18:0 ceramides, while the content of the proteins involved in glucose (GLUT4, glycogen synthase, hexokinase II, AKT) and lipid metabolism (adipose triglyceride lipase, fatty acid binding protein) were increased after HIIT. We demonstrate a higher content of saturated ceramide and diacylglycerol fatty acids in the muscle of older subjects compared to young. Moreover, the content of saturated ceramides was reduced and muscle glucose metabolism improved at protein level after HIIT. This study highlights an increased content of saturated ceramides in aging which could be speculated to influence insulin sensitivity.

## 1. Introduction

Physical function and metabolic health deteriorates with aging which increase the risk of disease [1,2,3]. Insulin sensitivity is frequently reduced with aging which is linked to reduced muscle mass and increased abdominal fat mass [4,5,6,7]. The influence and role of bioactive lipids for insulin resistance in humans are yet to be fully clarified [8,9], whereas in rodents and cell studies, the bioactive lipids ceramide and diacylglycerol (DAG) have been shown to induce adverse effects on insulin signaling in skeletal muscle [10,11,12]. In particular, ceramide and DAG subspecies containing long chain saturated fatty acids (FA) including C16:0 and C18:0 are indicated to be involved in insulin resistance [13,14]. However, it remains unclear whether age influence muscle bioactive lipid content and species composition independently [15,16,17,18,19]. In a cross-sectional study we observed a lower content of muscle C16:0, C18:0, total saturated ceramide FA and total ceramide with aging in man [20], while other studies have reported a higher content of specific saturated ceramide subspecies and total ceramide [16,21] or no difference [15] in old compared to young.

Endurance training has numerous effects including improved insulin sensitivity, body composition and cardiorespiratory capacity in human, but as a time consuming activity it may not fit well into a busy schedule of everyday life [1,22,23,24]. High-intensity interval training (HIIT) is a time-efficient alternative to endurance training which also improves body composition and cardiorespiratory capacity [25,26,27], but the effects on insulin sensitivity and metabolic health are not fully elucidated and even less is known about the effects of HIIT on ceramide and DAG in skeletal muscle. Richards et al. [28] and Robinson et al. [29] reported an improvement in insulin sensitivity in response to HIIT, while Arad et al. [30] found no effect. Considering the inconsistent data available on the role of ceramide and DAG in insulin resistance in humans we wanted to elucidate this area further. Therefore, the aim of this study was to investigate the influence of age and HIIT on ceramide and DAG content in skeletal muscle of overweight to obese subjects.

## 2. Results

### 2.1. Subject Characteristics

The young group had a higher body weight, body mass index (BMI), and lean body mass compared to the older, while body fat (%), total fat and visceral fat mass did not differ (Table 1). Glycated haemoglobin (HbA1c) and fasting glucose concentration were lower in the young group and fasting insulin concentration was higher whereas no difference was found in a Homeostatic Model Assessment of Insulin Resistance (HOMA-IR) when compared to the older group. Maximal oxygen uptake (VO_2_max) in ml·min^−1^ was higher in the young than the older group; however, no difference was seen when correcting for body weight. HIIT induced a significant reduction in body fat%, total fat and visceral fat mass and increased lean body mass, VO_2_max and the activity of citrate synthase (CS) and β-hydroxyacyl-CoA dehydrogenase (HAD) in muscle. HbA1c, fasting glucose and insulin concentration as well as HOMA-IR were not affected by the HIIT.

### 2.2. Muscle Lipids and Glycogen

The content of intramyocellular triglyceride (IMTG) and glycogen did not differ between the young and older subjects. The muscle glycogen content increased in response to HIIT, while IMTG content was unaffected (Table 1).

**Figure 1:**
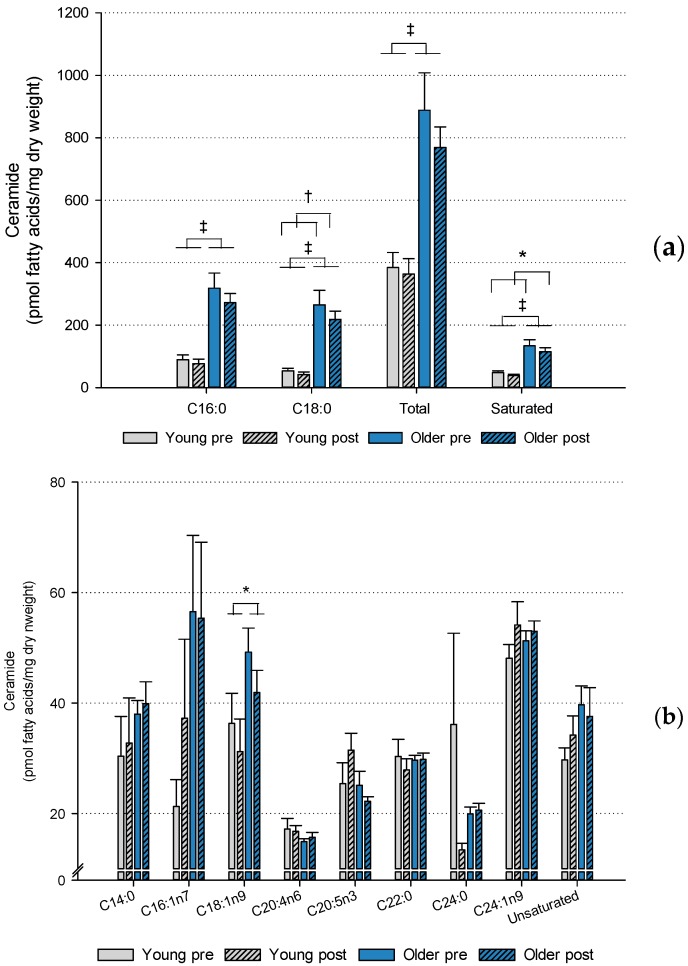
Ceramide content. The bar charts show the content of total and specific ceramide fatty acids in muscle of young and older subjects before and after 6 weeks’ high-intensity interval training. (**a**) Ceramide fatty acids of high abundance and (**b**) ceramide fatty acids of low abundance. Age and training effects: * *p* < 0.05, ^†^ *p* < 0.01, ^‡^ *p* < 0.001. Young: *n* = 12, older: *n* = 20. Data are means ± SEM.

There was an age effect on ceramide content in muscle with total ceramide (*p* < 0.001), saturated (*p* < 0.001), C16:0 (*p* < 0.001), C18:0 (*p* < 0.001) and C18:1 (*p* < 0.05) ceramide FA being higher in the older compared to the young subjects (Figure 1). Saturated ceramide FA (*p* < 0.05) and C18:0 (*p* < 0.01) were both reduced (main effect) in response to HIIT.The standard error mean of C16:1 and C24:0 ceramide, respectively, were relatively high which may be explained by individual differences between the subjects, and therefore, these data should be interpreted with caution (Figure 1b).

**Figure 2:**
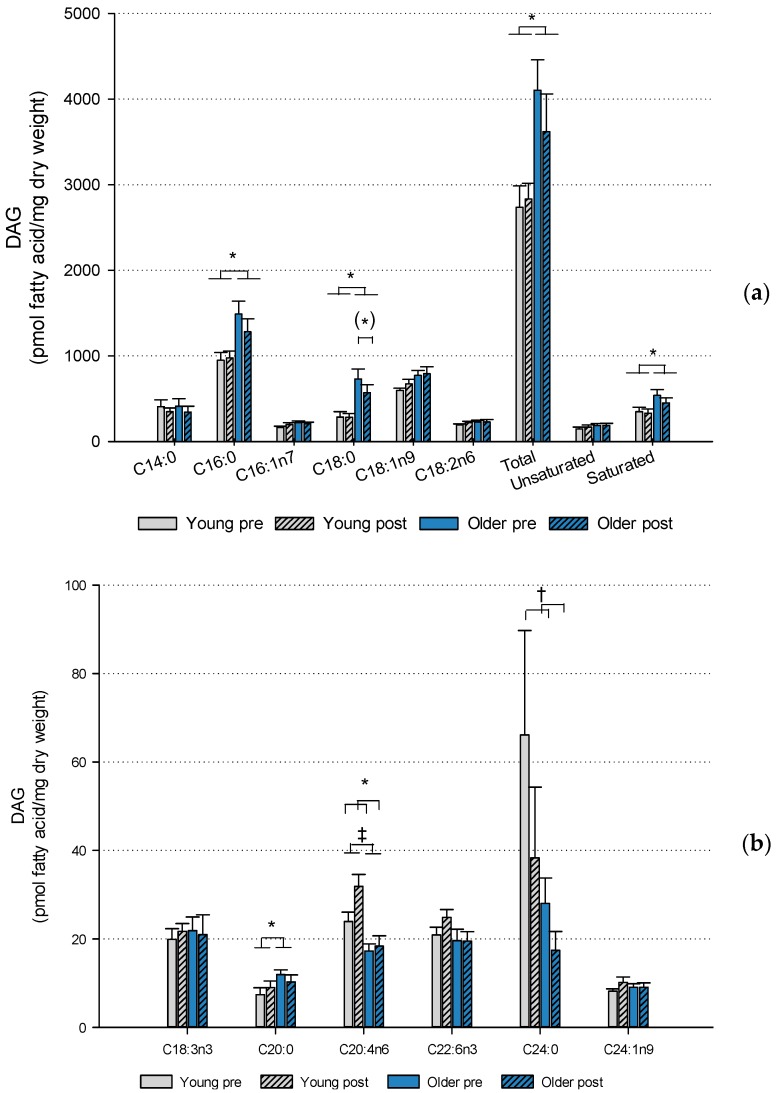
Diacylglycerol content. Bar charts illustrating the content of total and specific diacylglycerol fatty acids measured in muscle of young and older subjects before and after 6 weeks high-intensity interval training. (**a**) diacylglycerol fatty acids of high abundance and (**b**) diacylglycerol fatty acids of low abundance. Age and training effects: * *p* < 0.05, ^†^ *p* < 0.01, ^‡^ *p* < 0.001. Borderline significance: (*) *p* < 0.1. Young: *n* = 12, older: *n* = 21. Data are means ± SEM.

The older subjects had a higher content of total DAG FA (*p* < 0.05), saturated DAG FA (*p* < 0.05) and the saturated DAG FA C16:0 (*p* < 0.05), C18:0 (*p* < 0.05) and C20:0 (*p* < 0.05) in muscle compared to the young (Figure 2). The content of C20:4n6 DAG FA (*p* < 0.001) was higher in the young subjects, however the standard error mean is relatively high and the difference should be interpreted with caution (Figure 2b). A borderline significant (*p* = 0.055) interaction between age and training in C18:0 DAG FA showed a reduction (*p* = 0.023) in the older subjects in response to HIIT. Finally, HIIT training induced an increase in the content of C20:4n6 DAG FA (*p* = 0.05) whereas the C24:0 (*p* < 0.01) content was decreased.

Collectively, there was an effect of age and HIIT on the content of saturated DAG FA species measured and total DAG while several unsaturated species were not affected.

### 2.3. Protein Expression

**Figure 3:**
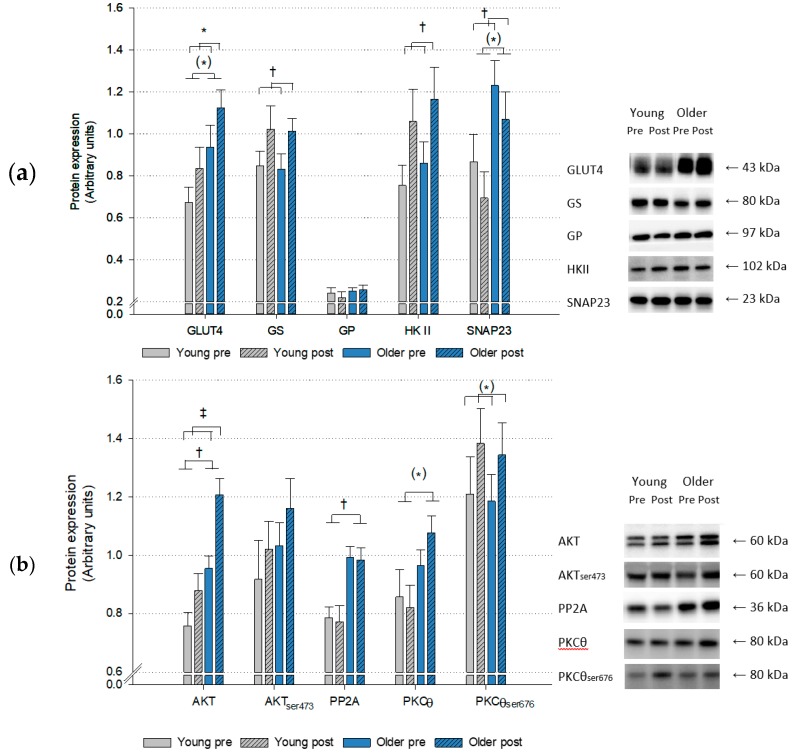

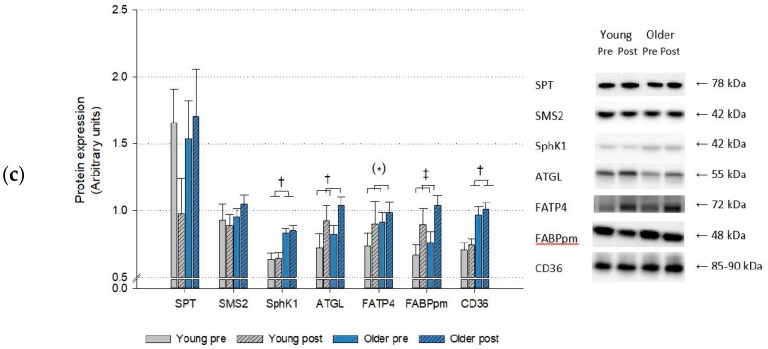
Protein expression in muscle of young and older subjects measured before and after 6 weeks high-intensity interval training. Expression of proteins involved in (**a**) glucose transport and metabolism, (**b**) insulin signalling and (**c**) ceramide and diacylglycerol (DAG) metabolism and lipid transport. Age and training effects: * *p* < 0.05, ^†^ *p* < 0.01, ^‡^ *p* < 0.001. Borderline significance: (*) *p* < 0.1. Young: *n* = 13, older: *n* = 21. Data are means ± SEM. AKT_ser473_: AKT phosphorylated at ser_473_, ATGL: Adipose triglyceride lipase, CD36: Cluster of differentiation 36, FABPpm: Fatty acid binding protein plasma membrane, FATP4: Fatty acid transport protein, GS: Glycogen synthase, GP: Glycogen phosphorylase, HKII: Hexokinase II, PKCθ: Protein kinase Cθ, PKCθ_ser676_: PKCθ phosphorylated at ser_676_, PP2A: Protein phosphatase 2A, SMS2: Sphingomyelin synthase 2, SphK1: Sphingosine kinase 1, SPT: Serine palmitoyl transferase, SNAP23: Synaptosome associated protein 23.

There was a higher expression of sphingosine kinase 1 (SphK1) (*p* < 0.01), AKT (*p* = 0.001), protein phosphatase 2A (PP2A) (*p* < 0.01) and cluster of differentiation 36 (CD36) (*p* < 0.01) and a borderline higher content of GLUT4 (*p* = 0.088), protein kinase Cθ (PKCθ) (*p* = 0.078) and synaptosome-associated protein 23 (SNAP23) (*p* = 0.071) in muscle of the older subjects compared to the young (Figure 3). HIIT induced an overall increase in protein expression of GLUT4 (*p* < 0.05), glycogen synthase (GS) (*p* < 0.001), hexokinase II (HK II) (*p* < 0.01), SNAP23 (*p* < 0.05), AKT (*p* < 0.001), fatty acid binding protein plasma membrane (FABPpm) (*p* < 0.001) and adipose triglyceride lipase (ATGL) (*p* = 0.001) and a trend towards an increase in fatty acid transporter protein 4 (FATP4) (*p* = 0.076) and PKCθ_ser676_ (*p* = 0.056) content. The content of glycogen phosphorylase (GP) was not influenced by either age or HIIT (Figure 3).

## 3. Discussion

In this study, an important observation was the higher content of total, saturated and C16:0 and C18:0 ceramide and DAG FA in muscle in older compared to young overweight to obese subjects. Whether the potentially more adverse composition of ceramide and DAG FA in the older subjects can be explained solely by aging or whether excess lipid availability in obesity interacts with aging is yet to be elucidated. Interestingly, HIIT induced an improvement in glucose metabolism, a reduction in total saturated ceramide FA, C18:0 ceramide FA and a trend towards a reduction in C18:0 DAG FA in muscle of the older subjects. Saturated ceramides are speculated to have adverse effects on insulin sensitivity; however, HOMA-IR, a surrogate measure of insulin resistance, remained unchanged after HIIT despite the reduced content of saturated ceramides.

A major finding of the present study was the higher content of total ceramide and DAG FA and several saturated species in muscle of older subjects compared to the young. Fat mass and IMTG measured in the young and older subjects were similar and can thus not explain this difference. Furthermore, the CS and HAD activities were similar between the young and older subjects indicating that the difference cannot be explained by a local muscle oxidative capacity difference. Bonen et al. [31] reported a higher content of CD36 in the muscle plasma membrane from both obese and subjects with type 2 diabetes compared to controls as well as a higher rate of transport of long chain FA into the muscle. In the present study the older subjects had a higher protein content of CD36 in muscle, and assuming this will lead to increased uptake of FA and thus higher intracellular long chain acyl CoA content, as demonstrated by Bonen et al. [31], this could favour ceramide and DAG accumulation.

Interestingly, we observed a higher protein content of SphK1 in the older compared to the young subjects, which may be stimulated through the higher total ceramide content, given that SphK1 contributes to removal of excess ceramide in muscle through the sphingosine pathway [32]. We did also measure SPT and SMS2 protein content, but observed no difference with age and, therefore, this does not provide an explanation for the mechanism leading to increased muscle ceramide content with age.

In the present study we found a higher content of saturated and C16:0 ceramide FA in the older subjects, which is concordant with previous findings in middle-aged and old subjects [16,21]. One study found that C18:0 ceramide was higher in 40- to 70-year-old obese insulin resistant subjects versus lean and obese insulin sensitive subjects [33] and combined with the presence of a negative association between insulin sensitivity and ceramide C16:0 and C18:0 content this indicates that long chain saturated ceramides may play a role in insulin resistance [13,14]. In support of this, the content of saturated and C18:0 ceramide FA was reduced in response to HIIT, and this supports a previous finding showing a reduced content of C18:0 ceramide in muscle of endurance trained athletes compared to patients with type 2 diabetes [14]. However, HOMA-IR was similar between the young and older groups, and although insulin sensitivity measured by HOMA-IR compared to the hyperinsulinemic-euglycemic clamp is less coupled to muscle insulin sensitivity, it is not possible to link the age induced higher saturated ceramide FA content to insulin sensitivity in this study. Furthermore, we did not observe an effect of HIIT on SphK1, SPT and SMS2 content, suggesting that the changes in ceramide content induced by HIIT, was not mediated by a HIIT induced change in these key regulatory proteins.

Bergman et al. [34] have reported significant inverse correlations between insulin sensitivity and total DAG, as well as C18:0/C18:0 DAG content [34]. In addition, Bergman and colleagues showed that C16:0/C16:0, C18:0/C18:0 and total DAG content were higher in the membrane in muscle of patients with type 2 diabetes when comparing to obese subjects and endurance trained athletes, which indicates a role for DAG in insulin resistance. Interestingly, we observed a higher content of total, saturated DAG FA and C16:0, C18:0 and C20:0 DAG FA in muscle of older compared to young subjects. Two studies found that PKCθ muscle protein content was increased in patients with type 2 diabetes and this implies that insulin signalling could be attenuated through activation by DAG [10,35]. The muscle protein content of PKCθ was higher in the older than the young subjects and this supports a possible link between long chain-saturated DAG species and attenuation of insulin signalling through PKCθ.

The present study demonstrates an effect of age with a higher total content of DAG FA and more of the distinct saturated species in the older group. The literature on age-induced alterations in DAG content is limited and characterized by inconsistency. Chee et al. [15] found a higher content of C18:1 DAG in obese old subjects versus old and young lean subjects, which is supported by Moro et al. [16] reporting that C18:1 DAG was higher in obese subjects compared to lean regardless of age. These data imply an effect of obesity rather than age on the DAG content and the specific content of C18:1 DAG. In contrast, Bergman et al. [34] found a higher muscle content of C18:1 DAG in athletes compared to controls. Moreover, Coen et al. [36] found no difference in DAG content in the muscle of obese elderly women whether insulin-sensitive or resistant. Albeit the effect of age on DAG FA in this study are interesting, there is a marked lack of consistency in the literature and further studies possibly studying specific subcellular locations are required to understand the possible coupling to insulin resistance.

Overall HIIT only significantly induced an increase in the content of C20:4n6 DAG FA. The content of C18:0/C18:0 and C16:0/C18:0 DAG subspecies have previously been found to be higher in endurance trained athletes compared to sedentary lean and obese subjects while moderate exercise was reported to reduce the total DAG and C16:0 DAG content [24,37,38,39]. Several studies observed no changes in DAG content in endurance trained versus untrained subjects or in response to moderate exercise and this further illustrate the discrepancy on the influence of training on DAG content [40,41,42].

The protein expression in muscle indicate that the capacity for glucose uptake were higher in the older subjects compared to young represented by a higher protein content of AKT and a trend towards higher GLUT4 content. However, no difference in content of activated AKT_ser473_ between groups was found which may be explained by the higher PP2A content in the older subjects which is a ceramide-activated protein suggested to dephosphorylate and hence inactivate AKT at residue ser473 [11]. This is in line with the higher content of ceramide and saturated species measured in the older subjects.

HIIT improved glucose metabolism in muscle reflected by increased AKT, GLUT4, HK II, SNAP23 and GS protein content as well as glycogen content, which imply an increased capacity for glucose uptake and storage in response to HIIT. The HIIT induced increase in GLUT4 content is strongly supported by previous studies [43,44,45]. HIIT however, did not induce any alterations in fasting plasma glucose concentrations, which is in line with other studies [46,47].

The observation of increased CS and HAD activity after HIIT are consistent with an improved oxidative capacity in muscle and increased utilization of lipid as preferable substrate [43,44]. Increased delivery of FA for lipid oxidation was reflected by an increased protein content of FABPpm and borderline FATP4. FABPpm and CD36 protein content has previously been shown to increase in response to HIIT [44] while another study showed no change in FABPpm or CD36 [45].

### Limitations

The comparison of young and older subjects in the present study has some limitations. Fewer subjects were included in the young group and the percentage of males was higher in young group compared to the older group. Male characteristics may therefore dominate in the young group within body composition, VO_2_max and metabolic parameters, and therefore a cautious comparison with the older group is required. The two groups were not BMI-matched which may also have influenced the results, but as the older group had a lower BMI, this should if anything rather attenuate a difference in bioactive lipid content and thus mask an age difference. The two studies in the young and older group were carried out in the same laboratory but separately, but the muscle analysis was performed together in randomized order eliminating day-to-day variation.

The lipid analysis detected the presence of ceramide and DAG FA and not the specific subspecies. Since ceramide only contain one FA, the data represent the presence of the ceramide subspecies but the combination of the two FA in DAG comprising the different subspecies was masked. The content of DAG FA was though measured indicating the availability for distinct DAG subspecies.

## 4. Methods and Materials

### 4.1. Subjects

Young (33 ± 1 years) and older (63 ± 1 years) subjects were recruited through advertisements in local newspapers. The inclusion criteria were age 20–40 or 55–75 years, Caucasian origin, BMI >27 kg/m^2^, sedentary (defined as <600 MET min/week using the International Physical Activity Questionnaire (IPAQ)). Only non-smokers were recruited and subjects with type 1 or 2 diabetes or metabolic or heart disease were excluded. All subjects had an electrocardiogram performed at the screening visit. All subjects gave informed written consent prior to their participation in the study. The study was approved by the Ethical Committee of Copenhagen (journal no. H-3-2012-024, permission date 4 February 2014) and complied with the guidelines of the Helsinki Declaration.

### 4.2. Study Design

In this longitudinal study, the subjects performed 6 weeks’ HIIT on a bicycle ergometer. During the first two weeks, prior to the HIIT intervention, different tests were carried out as described previously [48]. The hyperinsulinemic-euglycemic clamp was not performed in the young subjects and therefore these data measured in the older group will not be discussed here. On the first test day, body composition was determined by a dual energy X-ray absorptiometry scan (DXA) (Lunar iDXA, GE Healthcare, Madison, WI, USA) followed by an incremental maximal oxygen uptake (VO_2_max) test on a bicycle ergometer. On a second test day a muscle biopsy was obtained, fasting blood samples were collected and a second VO_2_max test performed. The tests were repeated after the HIIT intervention where the DXA scan was relocated to the first test day, which was placed 72 h after the last HIIT session.

Test days were separated by at least 48 h; 24 h prior to a test day, the subjects were asked not to perform vigorous exercise and to fast overnight. During their participation in the study, the subjects were informed to remain weight stable and not to change their physical activity level or diet.

### 4.3. High-Intensity Interval Training (HIIT) Protocol

The subjects performed 6 weeks supervised HIIT three times a week on a bicycle ergometer. The training load was determined during session 1, which consisted of up to 9 intervals where the load was increased with 10% at each interval starting at 85% of their maximal load measured at the VO_2_max test. The load at session 2–6 was equal to the load of the final interval completed at session 1 and it was increased by 10 % for the remaining sessions. A HIIT session consisted of 2 min warm up at 50 Watts followed by 5 intervals of 1 min HIIT with a cadence above 50 rounds per minute. The intervals were interrupted by 90 sec cycling at 25 Watts or just resting on the bike. Oxygen uptake was measured at session 6, 12 and 18 during the session and heart rate was monitored by a Polar T31 Transmitter, Finland, during each HIIT session.

### 4.4. Maximal Oxygen Uptake

An incremental test on a bicycle ergometer was performed to determine VO_2_max. The cycle was connected to a LODE Ergometry Manager computer program while a Cosmed online gas connecting system (Quark PFT Ergo, Cosmed, Rome, Italy) collected the data. Initially the subjects warmed up for 5 min at a 50 Watt load followed by 1 Watt increase in load every third sec until VO_2_max was achieved with the presence of a plateau in oxygen consumption and respiratory exchange ratio ≥1.15. Two VO_2_max tests were performed on two separate test days before the HIIT intervention to avoid that potential learning effects affected the data.

### 4.5. Blood Analyses

HbA1c was measured in a blood sample by a DCA Vantage Analyser (Tarrytown NY, USA). Fasting blood samples were obtained from a catheter placed in the dorsal vein of the hand. The drawn blood samples for analysis of plasma glucose were transferred to fluoride vacutainers (Cat. 368520, BD Albertslund, Denmark) and centrifuged at 1200× *g* for 1 min at room temperature. Blood samples for insulin analysis were transferred to pre cooled heparin vacutainers (Cat.367374) and centrifuged for 10 min at 2000× *g* and 4 °C. Plasma was transferred to eppendorf tubes after centrifugation and stored at −80 °C until analysed. Plasma glucose concentration was measured on a Hitachi Cobas 6000 (Roche A/S, Hvidovre, Denmark). Insulin concentration was assessed by commercial ELISA kits (ALPCO Diagnostics, Salem, HN, USA, cat. No. 80-INSHU-E01.1) and analysed on a Multiskan FC Microplate Photometer (Termo Fisher Scientific, Slangerup, Denmark).

### 4.6. Muscle Biopsies

Muscle biopsies were obtained in muscle vastus lateralis. The skin was sterilized and Lidocaine 5 mg/mL was injected to anesthetize the skin and the muscle fascia. The Bergstroem needle technique [49] was used to obtain the muscle biopsies. The biopsies were instantly divided, snap frozen in liquid nitrogen and transferred to eppendorf tubes. The muscle samples were stored at −80 °C for subsequent analyses.

Prior to analyses the muscle samples were cut and freeze dried for minimum 48 h at 0.5 mBar at −40 °C. Before dissection, the samples equilibrated to room temperature for one hour under controlled pressure and humidity and this was followed by dissection where visible fat, blood and connective tissue were removed.

### 4.7. Lipid Analyses

Ceramide and DAG total content and ceramide and DAG FA were measured in muscle. Approximately 20 mg muscle (wet weight) was dissected and lipids were extracted according to Folch [50] in the presence of internal standards (1,2-diheptadecanoyl-sn-glycerol and *N*-pentadecanoyl-d-erythro-sphingosine, Sigma). Ceramide and DAG were separated by thin-layer chromatography [51] by a resolving solution of diethyl ether: hexane: acetic acid (90:10:1; *v*:*v*:*v*). Lipid class standards were marked outside the chromatography plates and the corresponding gel bands were transferred to tubes and transmethylated in 14% methanolic boron trifluoride at 100 °C for 10 (DAG) or 90 (ceramide) minutes, respectively. The content of fatty acid methyl esthers was subsequently assessed by gas-liquid chromatography (Hewlett-Packard 5890 Series II with Varian CP-SIL 50 m × 0.25mm capillary column) [52].

### 4.8. Intramyocellular Triglyceride and Glycogen

IMTG was determined by lipid extraction, as previously described [50,53]. Glycogen content was measured by 2 h hydrolysis at 100 °C in 1 M HCL followed by determination of glycogen as glucose residues in a hexokinase based procedure [54].

### 4.9. Citrate Synthase and β-Hydroxyacyl-CoA Dehydrogenase

The activity of CS and HAD was measured in muscle by spectrophotometry (COBAS 6000, C 501, Roche Diagnostics, Mannheim Germany), as previously described [55].

### 4.10. Western Blot

To gain an understanding of the potential role of muscle ceramide and DAG in insulin resistance, expression of key proteins involved in ceramide and DAG metabolism and insulin signalling were measured. Western blotting was performed to measure protein expression in skeletal muscle as previously described [56] with modifications. In short, the dissected muscle samples were homogenized and protein concentration measured. Dilutions of the homogenate were made adding Laemmli buffer and MilliQ water to yield 10 µg protein per 10 µl homogenate; 5–10 µL of each sample were loaded on a 26 well 12% Criterion TGX Stain-Free polyacrylamide sodium dodecyl sulphate (SDS) gels and the proteins separated at 100–250 volt. The gels were activated with LAS 4000 image analyzer (GE Healthcare, Little Chalfont, UK) and 1 sec image was taken. The proteins were transferred to ethanol activated polyvinylidene fluoride (PVDF) membranes (0.2 µm pores, Bio-Rad, Copenhagen, Denmark) by 7 min semidry blotting at 25 V and a 1 sec ultraviolet (UV) light image taken of the membrane with the transferred proteins. The membranes were blocked in 2.5–5% skimmed milk or bovine serum albumin (BSA) diluted in Tris-buffered saline (TBS) or PBS and incubated with primary antibody at 4 °C over night. Primary antibodies: anti-GLUT4 1:12000 (PA1-1065, Fischer Scientific, Roskilde, Denmark), anti-glycogen synthase 1:4000 (#3893, Cell Signaling, Danvers, MA, USA), anti-glycogen phosphorylase (GP) 1:12000 (As09 455, Agrisera, Vännäs, Sweden), anti-hexokinase II (HKII) 1:1000 (ab104836, Abcam, Cambridge, UK) and anti-synaptosome associated protein (SNAP23) 1:3000 (ab3340, Abcam), anti-serine palmitoyl transferase (SPT) 1:4000 in 5% milk in TBS (ab23696, Abcam), anti-sphingomyelin synthase 2 (SMS2) 1:2000 in 5% milk in TBS (ab103060, Abcam), anti-sphingosine kinase 1 (SphK1) 1:1000 in 5% milk in TBS (ab37980, Abcam), anti-protein phosphatase 2A (PP2A) 1:1000 in 5% milk in TBS (ab32141, Abcam), anti-protein kinase C θ (PKCθ) 1:500 in 5% BSA in TBS (ab110728, Abcam), anti-protein kinase C θ p-ser676 (PKCθ_ser676_) 1:500 in 5% BSA in TBS (ab131479, Abcam), anti-fatty acid transport protein 4 (FATP4) 1:500 in 2.5% BSA in PBS (ab200353, Abcam), anti-fatty acid binding protein (FABPpm) 1:1000 in 5% milk in TBS (ab93928 [3E9], Abcam), anti-AKTpan (AKT) 1:1000 in 5% milk in TBS (#4691, Cell Signaling), anti-AKT p-ser473 (AKT_ser473_) 1:500 in 5% BSA in TBS (#4060, Cell Signaling), anti-CD36 1:3000 in 2.5% milk in TBS (AF1955, R&D Systems, Minneapolis, MN, USA), anti-adipose triglyceride lipase (ATGL) 1:500 in 2.5% milk in TBS (10006409, Cayman Chemical, Ann Arbor, MI, USA). Polyclonal goat anti-rabbit horseradish peroxidase (HRP) conjugated (7074S, Cell Signaling) was used as secondary antibody for GLUT4, GS, GP, HKII, SNAP23, PP2A, SphK1, SMS2, FATP4, FABPpm, AKT, AKT_ser473_, PKCθ, PKCθ_ser676_, ATGL (1:2000) and SPT (1:4000) while CD36 (1:3000) was incubated with polyclonal rabbit anti-goat HRP conjugated (P0449, DAKO, Santa Clara, CA, USA). The proteins were visualized and quantified as previously described [56] by normalizing the intensity of each band to total protein content measured by stain free fluorescence and samples on different gels were compared by quantification to a calibrator.

### 4.11. Statistical Analyses

Analysis of variance (ANOVA) with repeated measurements was used to analyse differences between the young and older subjects, and their response to 6 weeks HIIT. Holm–Sidak was used as the post hoc test. Data without normal distribution or equal variance were log transformed before performing the analysis. Sigmaplot 13.0 was used to perform the statistical analyses. Data are presented as means ± standard error of the mean (SEM).

## 5. Conclusions

This study demonstrates a higher content of ceramide and DAG, saturated FA and C16:0 and C18:0 ceramide and DAG FA in muscle in older overweight to obese compared to young subjects. The HOMA-IR was similar between groups despite the higher presence of these ceramide and DAG FA species that are coupled to insulin resistance. Whether the potentially more adverse composition of ceramide and DAG in the older subjects can be explained solely by aging or whether excess lipid availability in obesity interacts with aging is yet to be elucidated. Overall HIIT induced an improvement in glucose metabolism, a reduction in C18:0 ceramide FA, and a trend towards a reduction in C18:0 DAG FA in the muscle of the older subjects, but this did not lead to an improved HOMA-IR. However, despite these positive metabolic effects of HIIT, the key ceramide regulatory proteins remained unchanged and, therefore, this study failed to find a coupling to the muscle ceramide content. Further studies of longer duration are needed to clarify the potential influence of training on ceramide and DAG metabolism.

## Figures and Tables

**Table 1:** Subject characteristics.

	Young (*n* = 14) Pre Post		Older (*n* = 22) Pre Post		Main Effect (*p*-value) Age Time		Interaction (*p*-value) Group *x* Time
**Gender (F/M)**	5/9		11/11				
**Age (yrs)**	32 ± 2		63 ± 1				
**Height (m)**	1.78 ± 0.02		1.70 ± 0.02		0.014	NS	NS
**Weight (kg)**	110 ± 4	110 ± 4	88.7 ± 2.6	88.4 ± 2.6	<0.001	NS	NS
**BMI (kg·m^−2^)**	34.8 ± 1.0	34.6 ± 1.0	30.7 ± 0.7	30.6 ± 0.7	0.003	NS	NS
**LBM (kg)**	63.8 ± 2.1	64.7 ± 2.3	51.5 ± 2.1	51.8 ± 2.1	<0.001	<0.001	0.099
**Fat mass (kg)**	40.3 ± 3.1	39.3 ± 3.3	34.0 ± 1.6	33.3 ± 1.7	NS	0.016	NS
**Fat %**	39.2 ± 2.1	38.2 ± 2.3	39.8 ± 1.6	39.1 ± 1.6	NS	<0.001	NS
**Visceral fat (kg)**	1.67 ± 0.25	1.56 ± 0.24	1.90 ± 0.16	1.81 ± 0.16	NS	0.024	NS
**HbA1c (%)**	5.3 ± 0.1	5.3 ± 0.1	5.7 ± 0.1	5.6 ± 0.1	0.002	NS	NS
**HOMA-IR (AU)**	2.14 ± 0.24	2.31 ± 0.38	1.88 ± 0.23	1.99 ± 0.30	NS	NS	NS
**Glucose, fasting (mmol·L^−1^)**	4.5 ± 0.1	4.5 ± 0.1	6.1 ± 0.2	6.0 ± 0.2	<0.001	NS	NS
**Insulin, fasting (pmol L^−1^)**	69.7 ± 9,5	67.2 ± 8.9	40.9 ± 4.8	42.6 ± 5.8	0.008	NS	NS
**IMTG (mmol·kg^−1^ dw)**	126 ± 27	118 ± 21	156 ± 23	119 ± 12	NS	NS	NS
**Glycogen (nmol·kg^−1^ dw)**	236 ± 30	474 ± 46	323 ± 24	483 ± 23	NS	<0.001	NS
**HAD (µmol·g^−1^·min^−1^)**	116 ± 7	130 ± 7	112 ± 10	141 ± 5	NS	<0.001	NS
**CS (µmol·g^−1^·min^−1^)**	132 ± 7	165 ± 8	122 ± 10	169 ± 10	NS	<0.001	NS
**VO_2_max (mL·min^−1^)**	3068 ± 131	3186 ± 118	2234 ± 106	2361 ± 134	<0.001	0.021	NS
**VO_2_max (mL·min^−1^·kg^−1^)**	28.3 ± 1.2	29.7 ± 1.5	25.2 ± 1.0	26.7 ± 1.1	NS	0.007	NS

Characteristics of young and older subjects before and after 6 weeks high-intensity interval training. IMTG analysis: Young: *n* = 2F/8M. AU: Arbitrary unit, BMI: body mass index, CS: Citrate synthase, dw: dry weight, HAD: β-hydroxyacyl-CoA dehydrogenase, HOMA-IR: homeostatic assessment model of insulin resistance, IMTG: intramyocellular triglyceride, LBM: lean body mass. Data are means ± standard error of the mean (SEM).
